# Centrifugal Atomization and Characterization of Fe-Si-B Amorphous Alloys

**DOI:** 10.3390/ma18030510

**Published:** 2025-01-23

**Authors:** Sasha A. Cegarra, Héctor Maicas, Jordi Pijuan

**Affiliations:** 1 Eurecat, Centre Tecnològic de Catalunya, Unit of Metallic and Ceramic Materials, Plaça de la Ciència 2, 08243 Manresa, Spain; sasha.cegarra@eurecat.org (S.A.C.); hector.maicas@eurecat.org (H.M.); 2 Departament d’Enginyeria Industrial i de l’Edificació, Universitat de Lleida, Jaume II 69, 25003 Lleida, Spain

**Keywords:** centrifugal atomization, metallic glasses, cooling rate, amorphous fraction, amorphous alloy

## Abstract

The centrifugal atomization process is a rapid solidification method that achieves high cooling rates. Although this technique is typically used to produce common metal powders, it has not been extensively explored for amorphous powder production, despite its clear advantage of generating nearly perfect spherical particles, which is beneficial for subsequent powder consolidation. In this paper, a characterization of three iron-based alloys from the Fe-Si-B system, specifically Fe_91.72_Si_5.32_B_2.96_ (wt%), Fe_87.37_Si_6.94_B_2.49_Cr_2.46_C_0.75_ (wt%), and Fe_89.41_Si_2.02_B_1.13_P_5.89_C_1.55_ (wt%), produced by centrifugal atomization, is presented. The amorphous fractions of the powders were quantified using DSC, with further characterization performed via optical microscopy, SEM, and XRD. The amorphous fractions increased with the addition of Cr, C, and P, reaching up to 90% in the Fe_89.41_Si_2.02_B_1.13_P_5.89_C_1.55_ alloy for particles of <100 μm. The onset cooling rates were estimated to be approximately 10⁶ K/s for Fe_91.7_Si_5.32_B_3_, 10⁵ K/s for Fe_87.36_Si_6.9_B_2.48_Cr_2.45_C_0.75_, and 10⁴ K/s for Fe_89.41_Si_2.02_B_1.13_P_5.89_C_1.55_, respectively.

## 1. Introduction

Fe-based metallic glasses have gained significant interest due to their exceptional strength, soft magnetic properties, and corrosion resistance [1,2]. The development of the first iron-based bulk metallic glass was reported in the 1990s by Inoue [3], which led to continuous research on this material system, leading to numerous industrial applications such as magnetic parts for power transformers or magnetic-shielding sheets, inductors, and electrical machines, among others [4,5].

Typically, these metallic glasses are fabricated through melt spinning, as this method provides the necessary cooling rates for achieving metallic glass formation [6]. However, melt spinning has limitations in terms of the thickness of the resulting material, which is why producing these alloys in powder form presents a promising method for subsequent consolidation via additive manufacturing. In recent years, powder processing through additive manufacturing (AM) techniques has opened up new opportunities for processing and consolidating metallic glasses [5,7,8,9]. However, it is essential to assess powder characteristics such as morphology, particle size and distribution, flowability, bulk properties, and porosity to ensure optimal performance in the consolidation process and high quality in the final structures [10]. In this sense, the technique used to produce the powder plays a crucial role.

Metallic glass powders are usually prepared by mechanical alloying or atomization techniques. In mechanical alloying, materials are cold welded and diffused to produce amorphous powder via high-energy ball milling [11,12]. However, powders prepared by these methods generally have irregular structures, which is not ideal for subsequent consolidation. Atomization techniques include water and gas atomization methods, whereby the liquid metal is broken up by a high-pressure jet of inert gas or water, and the resulting droplets cool and solidify in the form of powder [13,14,15]. The cooling rates achieved by these techniques range from 10^2^ to 10^5^ K/s, which are adequate for producing amorphous powders. However, water atomization does not guarantee a spherical shape of the particles in the atomized powders, and water can react with the melt, creating oxides. Gas atomization, although it can produce high-quality powders, is an energy-intensive method, due to the use of high-pressure inert gas in the atomization process.

In this context, centrifugal atomization emerges as an efficient process for the production of metallic glasses, resulting in nearly perfectly spherical particles [16]. This process has been previously tested for the atomization of Al-based metallic glasses, but has not been studied for the production of Fe-based metallic glass [17]. This technique involves a stream of liquid metal being dropped onto a rapidly spinning disc, whereby the centrifugal force breaks the droplets, which then solidify in the form of powder [18,19]. This process achieves high cooling rates up to 10^5^ K/s, and produces small particles in the micron range [17,20].

In the development of iron-based metallic glasses, the Fe-Si-B system has been frequently studied, due to its high magnetic saturation, lack of intrinsic magnetocrystalline anisotropy, and low cost [21,22]. Moreover, the addition of C to the Fe-Si-B system can enhance the alloy’s glass-forming ability, while maintaining a high saturation magnetic flux density, as well as enhancing the thermal stability [23,24]. On the other hand, adding Cr to the system can increase electrical resistivity and improve corrosion resistance [25]. Finally, adding P can effectively improve the glass-forming ability, the stability of the supercooled liquid region, and the corrosion resistance [26].

In this study, three different alloy compositions were selected with which to investigate the centrifugal atomization process for producing Fe-amorphous powders. These were (1) Fe_91.72_Si_5.32_B_2.96_ (wt%), (2) Fe_87.37_Si_6.94_B_2.49_Cr_2.46_C_0.75_ (wt%), and (3) Fe_89.41_Si_2.02_B_1.13_P_5.89_C_1.55_ (wt%). These alloys were atomized using centrifugal atomization, and the microstructure and amorphous fraction of each composition were studied. These specific alloys were chosen for their glass-forming ability, which has been explored using other methods, but not through centrifugal atomization [9,27,28,29]. The aim of this research is to characterize the glass-forming ability of these compositions, and to evaluate and expand the potential of producing them via centrifugal atomization, taking advantage of the benefits that this method offers.

## 2. Materials and Methods

### 2.1. Powder Production

The alloy compositions studied in this work are (1) Fe_91.72_Si_5.32_B_2.96_ (wt%), (2) Fe_87.37_Si_6.94_B_2.49_Cr_2.46_C_0.75_ (wt%), and (3) Fe_89.41_Si_2.02_B_1.13_P_5.89_C_1.55_ (wt%). For the sake of simplification and to avoid repetition, these alloys will henceforth be referred to as FeSiB, FeSiBCrC, and FeSiBPC, respectively.

Commercial raw materials were used for alloy production. An ARMCO^®^ Pure Iron rounded bar served as the base material. Silicon, boron, phosphorus, and chromium were added using commercial ferroalloys: Fe-75%Si, Fe-18%B, Fe-25%P, and Fe-70%Cr, respectively. For carbon addition, pure graphite powder was used.

A centrifugal atomizer was used to produce the set of Fe-amorphous metal powders. Further details of the equipment are available in [20,30]. Raw materials were mixed in their respective proportions to produce each composition, with a total mass of 1 kg designed for each batch. The raw materials were melted using an induction heating system up to 1873 K, and were maintained at this temperature for 10 min to ensure good homogenization of the melt. Subsequently, the melt was poured onto a spinning disc 40 mm in diameter, provided with a ceramic coating, which was rotating at 35,000 rpm. The melting and atomization process was performed within an atomization chamber which had been vacuumed at 10^−3^ Pa and filled with He gas, ensuring an inert gas atmosphere throughout the process.

### 2.2. Powder Characterization

Each powder sample collected from the atomizer underwent initial sieving, according to ASTM-B15, for 15 min, with a Filtra FTL-0150 electromagnetic digital sieving machine (Filtra Vibracion SL, Badalona, Spain) with 45, 75, 106, 150, and 355 μm sieves. The chemical composition of each sample was studied using inductively coupled plasma optical emission spectroscopy (ICP-OES), using a Thermo Scientific iCAP Pro (Thermo Fisher Scientific Inc., Waltham, MA, USA). X-ray diffraction (XRD) (Bruker AXS GmbH, Karlsruhe, Germany) measurements were performed with a Bruker AXS D8 Advance to identify crystalline phases. The samples were run and analyzed from 40 to 100° (2-theta), with a step size of 0.020°. Differential Scanning Calorimetry (DSC) was performed on selected samples to calculate the amorphous fractions. DSC measurements were conducted, using a Mettler Toledo DSC1 (Mettler Toledo, Greifensee, Switzerland), on 20 mg samples, which were heated to a maximum temperature of 1473.15 K, at a heating rate of 10 K/min, under an Ar atmosphere.

Particle shape and microstructure were analyzed using an Epiphot 200 Light Optical Microscope (LOM) (Nikon Corporation, Tokyo, Japan) and an Ultra Plus field emission scanning electron microscope (SEM) (Carl Zeiss AG, Oberkochen, Germany). For microstructural observation, the powder samples were embedded in epoxy resin using standard metallographic methods for uncompacted metal powders [31]. Subsequently, the samples were ground with SiC paper and polished using various diamond suspensions up to 1 μm in size. Finally, the samples were etched with Nital (5% nitric acid in ethanol) for approximately 1 min, to reveal microstructural details.

## 3. Results and Discussion

The final compositions of the alloys are shown in Table 1. The Cr and C contents are well-adjusted across all alloys. However, a significant reduction in B content can be observed compared to the expected values for all compositions. Additionally, the Si content varies slightly in the FeSiBCrC alloy. These variations may be attributed to the melting strategies used for the ferroalloys and improper melting of the raw material.

### 3.1. SEM Analysis

**Figure 1:**
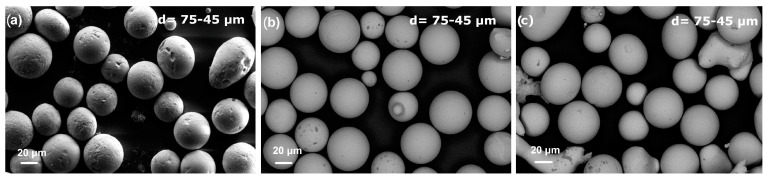
The surface morphology of the resulting powders for the three different compositions, in the size range of 75–45 µm: (**a**) Fe_91.72_Si_5.32_B_2.96_, (**b**) Fe_87.37_Si_6.94_B_2.49_Cr_2.46_C_0.75_, and (**c**) Fe_89.41_Si_2.02_B_1.13_P_5.89_C_1.55_.

Figure 1 shows the SEM morphology of the centrifugally atomized powders, sized between 75 and 45 μm, for the three compositions studied. Most of the powders are spherical, with only a minor presence of irregular shapes. The images of the powders of the compositions FeSiBCrC and FeSiBPC show a smoother surface than powder of the composition FeSiB, suggesting that the FeSiBCrC and FeSiBPC powders may contain some amorphous fraction, while the FeSiB powder likely reveals the presence of a crystalline microstructure.

### 3.2. Effect of Composition

**Figure 2:**
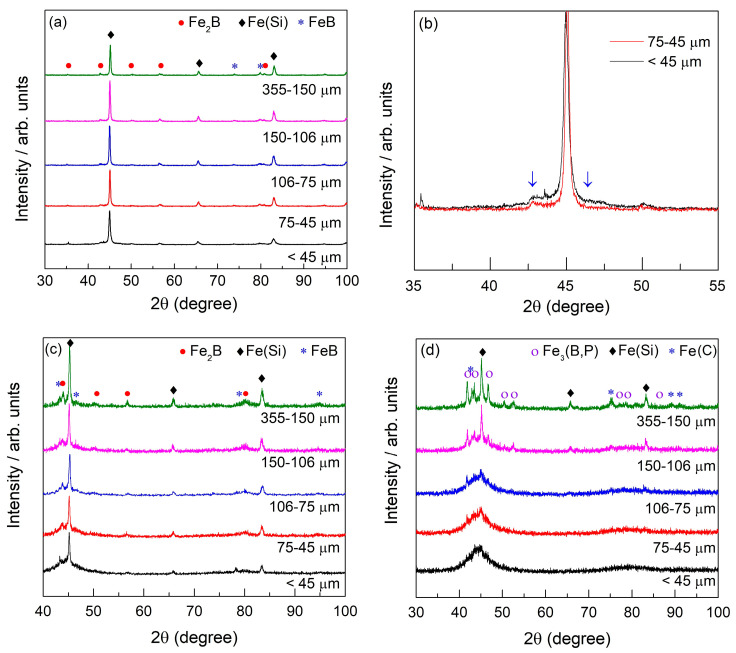
The XRD patterns of the centrifugally atomized powder samples for the three compositions studied: (**a**) Fe_91.72_Si_5.32_B_2.96_, (**b**) zoomed-in XRD pattern of Fe_91.72_Si_5.32_B_2.96_ for the <45 and 75–45 µm particle size ranges, (**c**) Fe_87.37_Si_6.94_B_2.49_Cr_2.46_C_0.75_, and (**d**) Fe_89.41_Si_2.02_B_1.13_P_5.89_C_1.55_.

Figure 2 shows the XRD patterns of the three compositions studied. In Figure 2a, the XRD pattern for the FeSiB alloy, across all particle size ranges, displays strong diffraction peaks. The primary crystalline phase is a bcc Fe-rich phase, which is a solid solution primarily containing Si, with stronger peaks appearing at 45°, 65.60°, and 83°, respectively. The secondary crystalline phases are minor, and correspond to borides, specifically FeB and Fe_2_B. In Figure 2b, a zoomed-in view of the XRD patterns for this alloy in the <45 and 75–45 µm particle size ranges is presented, highlighting a distinct difference in patterns, shown by the blue arrows. For the <45 µm range, a small halo is observed compared to the 75–45 µm range, indicating the presence of a partially amorphous structure.

The FeSiBCrC sample displays a diffraction pattern that is more typical of an amorphous powder, characterized by broader halos and fewer distinct crystalline peaks, indicating a higher amorphous fraction. However, strong diffraction peaks can still be observed, particularly for the bcc Fe(Si) phase, with prominent reflections at 45.24°, 66°, and 83.5°. Additionally, there is a minor presence of intermetallic compounds, specifically Fe_2_B and FeB.

For the FeSiBPC alloy, the XRD pattern reveals the characteristic halo of an amorphous phase, indicating a very high proportion of amorphous content. Up until 106 µm, the sample shows a broad halo. For higher particle sizes, crystalline phases clearly start to appear. For the 355–150 µm sample powder, the crystalline phase is in high proportion. The most significant crystalline phase that appears is bcc-Fe(Si) with Si in solid solution. An fcc-Fe(C) phase and an Fe_3_(B,P) intermetallic phase are also detected.

**Figure 3:**
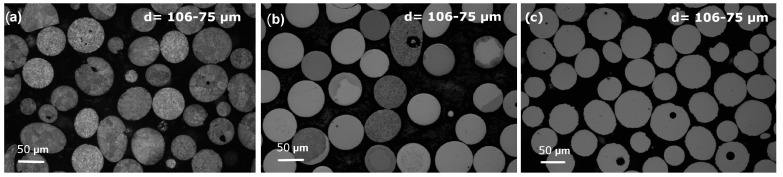
Optical microscope micrographs of the cross sections of the centrifugally atomized powder samples for the three compositions studied: (**a**) Fe_91.72_Si_5.32_B_2.96_, (**b**) Fe_87.37_Si_6.94_B_2.49_Cr_2.46_C_0.75_, and (**c**) Fe_89.41_Si_2.02_B_1.13_P_5.89_C_1.55_.

A comparison between the XRD results in Figure 2 and the micrographs shown in Figure 3, which depict the three compositions studied in the 106–75 μm particle size range, reveals a strong correlation between the two analyses. For the FeSiB alloy, both the XRD pattern and the micrographs confirm a fully crystalline structure. In contrast, the addition of Cr and C to the system (FeSiBCrC alloy) results in partially amorphous particles, as observed optically and supported by the broader halos between 40° and 50° in the XRD pattern. Moreover, the FeSiBPC alloy, which includes P and C, exhibits an optically featureless structure, corresponding to the amorphous phase indicated by the characteristic halo in the XRD analysis. These results confirm that the XRD findings and optical micrographs are in agreement.

Various criteria have been proposed for assessing GFA, and these criteria often depend on the alloy’s composition [32]. According to Inoue’s criterion [33], which emphasizes the significance of atomic size differences among alloying elements, the order from largest to smallest atomic radius is Cr > Fe > Si > P > B > C. Table 2 displays these atomic sizes and their mismatch with Fe atoms. Notably, carbon (C) has the smallest atomic radius and the greatest mismatch with Fe, followed by boron (B), which can improve atomic packing and, consequently, GFA. In contrast, chromium exhibits minimal mismatch with Fe, while phosphorus has a larger mismatch than chromium. Therefore, based on atomic size differences, the FeSiBPC alloy demonstrates the best GFA among the studied compositions.

### 3.3. DSC Analysis and Determination of Amorphous Fraction

**Figure 4:**
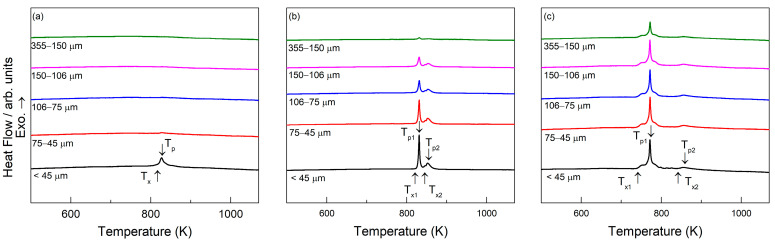
DSC curves for the three centrifugally atomized compositions for all the size ranges studied: (**a**) Fe_91.72_Si_5.32_B_2.96_, (**b**) Fe_87.37_Si_6.94_B_2.49_Cr_2.46_C_0.75_, and (**c**) Fe_89.41_Si_2.02_B_1.13_P_5.89_C_1.55_. T_x_ is the onset temperature of crystallization. T_p_ is the peak temperature of crystallization.

The DSC traces for the three Fe-based glass-forming alloys are shown in Figure 4, across five particle size ranges: 20–45 µm, 45–75 µm, 75–106 µm, 106–150 µm, and 150–355 µm. All the compositions exhibit crystallization peaks, confirming the presence of an amorphous phase, especially with smaller particle sizes. As particle size increases, the DSC peaks become less pronounced, indicating a higher degree of crystallinity. Table 3 summarizes the crystallization temperatures and the enthalpy of crystallization for the three studied alloys. For the FeSiB alloy, a crystallization event occurs in the 45 µm size range, starting at T_x_ = 819.45 K, which is consistent with values reported in the literature [34,35]. No crystallization peaks can be detected for the larger particle size ranges, confirming that the structure is fully crystalline at larger sizes.

In the case of the FeSiBCrC alloy, a primary crystallization event can be identified, followed by a smaller secondary event, with both contributing to the total enthalpy released during crystallization. The crystallization peaks remain well defined up to the 150–106 µm particle size range, indicating the presence of an amorphous phase within particles of this size. However, beyond this range, the intensity of the peaks decreases. The primary crystallization event begins at T_x1_ = 828.25 K, while the secondary event starts at T_x2_ = 843.51 K, with both values aligning with previously reported data [9].

For the FeSiBPC alloy, a single crystallization event is observed, beginning at T_x_ = 738.44 K. Notably, the crystallization peak is evident for particle sizes up to the 355–150 µm range, suggesting that the glass-forming ability of this alloy is superior to that of the other two alloys. This extended stability of the amorphous phase at larger particle sizes shows the improved glass-forming ability of FeSiBPC compared to the FeSiB and FeSiBCrC alloys.

These DSC curves align with the XRD analysis, showing that the FeSiB alloy predominantly consists of crystalline phases. The FeSiBCrC alloy exhibits a certain degree of amorphicity, while the FeSiBPC alloy displays the highest level of amorphicity, as evidenced by both the DSC and XRD results. In FeSiBPC amorphous alloys, increasing the phosphorus (P) content enhances glass-forming ability (GFA).

**Figure 5:**
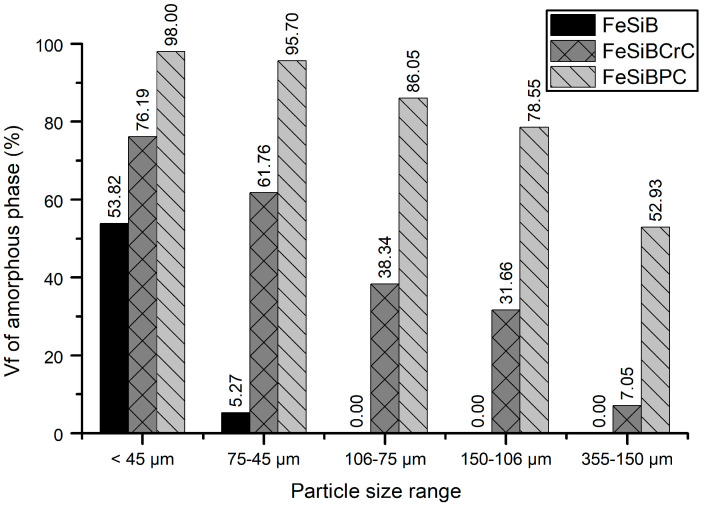
The amorphous volume fractions of the three centrifugally atomized compositions for all the size ranges studied.

Henao [28] et al. suggest using the crystallization energy of a fully amorphous melt-spun ribbon as a reference for determining the amorphous fraction in synthesized particles. Adopting this approach, we utilized literature data to analyze our centrifugally atomized powders for the FeSiB and FeSiBCrC alloys [28,37]. However, for the FeSiBPC alloy, a comparison was conducted with a sample with a particle size of less than 45 µm, which was estimated to be approximately 98% amorphous, as supported by XRD and SEM analyses, described in Section 3.1 and Section 3.2. This approach was chosen due to the lack of literature references for the crystallization energy of fully amorphous melt-spun ribbons or powders for this specific alloy. Figure 5 shows the amorphous fractions of the three alloys, determined by the crystallization enthalpies for the different particle size ranges studied in this article.

In line with the XRD results, the FeSiB alloy exhibited the lowest amorphous fraction, while FeSiBPC alloy achieved the highest. As anticipated, smaller particle sizes of less than 45 µm resulted in a higher amorphous fraction, with the FeSiBPC alloy reaching 98% amorphous content, compared to 53% for the FeSiB alloy. The alloy that attained the highest amorphous fraction was the FeSiBPC alloy, which achieved up to 53% amorphous content in the 355–150 μm particle size range. These results confirm that adding Cr and C to the Fe-Si-B system enhances the glass-forming ability of Fe-based metallic glasses. Furthermore, the addition of P further improves this ability, enabling the achievement of high amorphous fractions even with large particle sizes up to 355 μm.

### 3.4. Effects of Cooling Rate

To calculate the cooling rate of the atomized particles, a heat transfer model was employed. The details of the model are described in [17]. The model allows for the calculation of the cooling time of a droplet, based on the energy transferred by convection and radiation between the surface of the droplet and the surrounding environment. The heat transfer coefficient was determined using the Nusselt number from Whitaker’s semi-empirical correlation [38]. The cooling rate was defined as the time during which primary crystallization occurred, specifically, the time between the liquid temperature of the atomized melt T_l_ and the glass transition temperature of the corresponding alloy T_g_.

**Figure 6:**
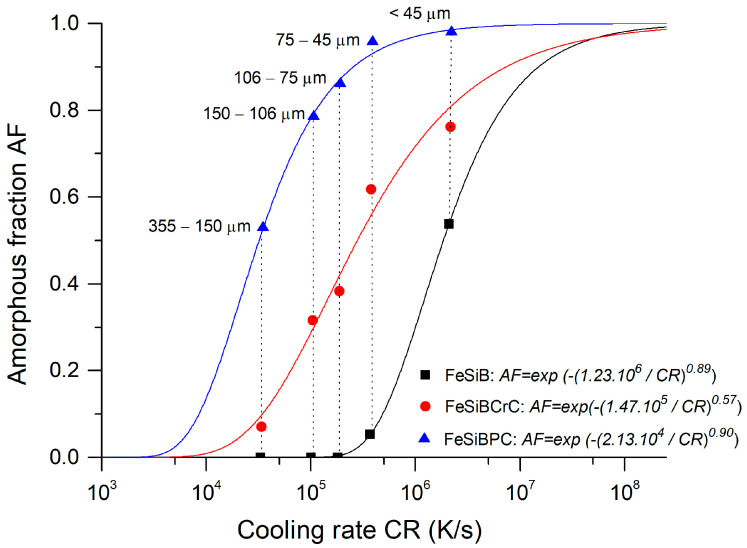
The amorphous fractions obtained for each composition, compared to the calculated cooling rates for the different particle sizes studied. The data points represent the particle sizes.

Figure 6 shows the amorphous fraction as a function of the calculated cooling rate for different particle sizes. The cooling rates range from 10⁴ K/s for larger particles, to 10⁶ K/s for smaller ones. The graph illustrates that as the cooling rate increases, the amorphous fraction also increases, highlighting the differences between the three alloys. At a fixed cooling rate, the amorphous fraction progressively rises from the FeSiB alloy to the FeSiBPC alloy. The highest amorphous fraction is achieved by the Fe_89.41_Si_2.02_B_1.13_P_5.89_C_1.55_ alloy with particles of 22.5 μm, at a cooling rate of 2.22·10⁶ K/s.

An exponential fit, using an exponential equation in the form of AF = exp(-(A/CR)^B^), was used to describe the amorphous fraction (AF) content as a function of the cooling rate (CR). A and B are the fitted parameters. Parameter A is interpreted as the threshold cooling rate at which amorphous structures begin to form in the system, marking the onset of amorphicity. Based on this fitting, the onset cooling rate values are 1.23·10^6^ for FeSiB, 1.47·10^5^ for FeSiBCrC, and 2.13·10^4^ for FeSiBPC. These values fall within the range of cooling rates reported for Fe-based metallic glasses with similar compositions, particularly those produced by gas and water atomization techniques [39]. Parameter B describes the width of the transition region between a fully amorphous sample and a fully crystalline sample. The coefficients of determination, R^2^, of the regression equations are above 0.96 in the three cases, indicating an adequate fit of the model.

The theoretical cooling rate values calculated for each particle size range, combined with the onset cooling rate for amorphization, demonstrate the high cooling capabilities of the centrifugal atomization process. This is further evidenced by the significant percentage of amorphous powder obtained across the various particle sizes. These results highlight that centrifugal atomization can achieve sufficiently high cooling rates to produce amorphous fractions that are comparable to those achieved by other atomization methods, such as gas and water atomization [15,39].

## 4. Conclusions

Three Fe-based amorphous alloys were successfully produced by centrifugal atomization: (a) Fe_91.72_Si_5.32_B_2.96_, (b) Fe_87.37_Si_6.94_B_2.49_Cr_2.46_C_0.75_, and (c) Fe_89.41_Si_2.02_B_1.13_P_5.89_C_1.55_, representing a significant achievement, as this method has not previously been demonstrated to produce amorphous structures in these alloy systems. The amorphous fractions were quantified using DSC, revealing that the Fe_89.41_Si_2.02_B_1.13_P_5.89_C_1.55_ alloy achieved the highest amorphous fraction of up to 90% for particles less than 106 µm in size, followed by Fe_87.37_Si_6.94_B_2.49_Cr_2.46_C_0.75_, with an amorphous fraction of 60% for particle sizes up to 75 µm, while Fe_91.72_Si_5.32_B_2.96_ remained mostly crystalline, except for with particle sizes less than 45 µm. This was further confirmed by XRD and SEM analysis, demonstrating that the addition of Cr, C, and P to the Fe-Si-B system significantly enhanced the glass-forming ability of these amorphous alloys.

A theoretical heat transfer model was used to evaluate the theoretical cooling rates of the atomized particles, and an exponential correlation between the experimental results of the amorphous fractions and the cooling rates was found. The estimated onset cooling rate values for the atomized alloy powders were 1.23·10^6^ for the Fe_91.72_Si_5.32_B_2.96_ alloy, 1.47·10^5^ for the Fe_87.37_Si_6.94_B_2.49_Cr_2.46_C_0.75_ alloy, and 2.13·10^4^ for the Fe_89.41_Si_2.02_B_1.13_P_5.89_C_1.55_ alloy, demonstrating the process’s capability to achieve sufficiently high cooling rates to obtain high amorphous fractions.

Importantly, the centrifugal atomization process has been shown to achieve the high cooling rates necessary for amorphous structure formation, thus expanding its application potential.

## Figures and Tables

**Table 1:** The resulting chemical compositions (wt%) of the three studied alloys after atomization.

	Fe %	Si %	B %	P %	Cr %	C %
FeSiB	bal.	5.2	1.51	-	-	-
FeSiBCrC	bal.	7.2	1.28	-	2.71	0.75
FeSiBPC	bal.	1.8	0.5	4.39	-	1.45

**Table 2:** The atomic radii of the elements (r_c_) and their atomic size mismatches with the Fe atom (r_Fe_ – r_x_), where x represents the corresponding element.

Element	Fe	Cr	Si	P	B	C
*r_c_* (*Å*)	1.25	1.28	1.18	1.10	0.82	0.77
*r_Fe_ – r_x_*	-	0.03	0.07	0.15	0.43	0.48

**Table 3:** Crystallization temperatures, T_x1_ and T_x2_, and enthalpies of crystallization, ΔH_1_ and ΔH_2_, for the three studied alloys, corresponding to particles sizes < 45 μm.

Powder Sample	T_g_ (K)	T_x1_ (K)	T_x2_ (K)	T_p1_ (K)	T_p2_ (K)	ΔH_1_ (J/g)	ΔH_2_ (J/g)
Fe_91.7_Si_5.32_B_3_ (<45 μm)	693.15	819.45	-	826.79	-	47.95	-
**Fe_87.36_Si_6.9_B_2.48_Cr_2.45_C_0.75_ (<45 μm)**	749	828.25	843.51	831.18	852.67	67.65	27.58
Fe_89.41_Si_2.02_B_1.13_P_5.89_C_1.55_ (<45 μm)	-	738.44	-	839.54	-	90.67	7.2
T_g_ values were obtained from the literature: FeSiB [36] and FesiBCrC [28]							

## Data Availability

The original contributions presented in this study are included in the article. Further inquiries can be directed to the corresponding author.
